# Deciphering the role of Epstein-Barr virus in the pathogenesis of T and NK cell lymphoproliferations

**DOI:** 10.1186/2042-4280-2-8

**Published:** 2011-09-07

**Authors:** Christopher P Fox, Claire Shannon-Lowe, Martin Rowe

**Affiliations:** 1 University of Birmingham College of Medical and Dental Sciences, School of Cancer Sciences, Edgbaston, Birmingham, B15 2TT, UK; 2 Nottingham University Hospitals, Department of Clinical Haematology, City Hospital campus, Hucknall Road, Nottingham, NG5 1PB, UK

## Abstract

Epstein-Barr virus (EBV) is a highly successful herpesvirus, colonizing more than 90% of the adult human population worldwide, although it is also associated with various malignant diseases. Primary infection is usually clinically silent, and subsequent establishment of latency in the memory B lymphocyte compartment allows persistence of the virus in the infected host for life. EBV is so markedly B-lymphotropic when exposed to human lymphocytes *in vitro *that the association of EBV with rare but distinct types of T and NK cell lymphoproliferations was quite unexpected. Whilst relatively rare, these EBV-associated T and NK lymphoproliferations can be therapeutically challenging and prognosis for the majority of patients is dismal. In this review, we summarize the current knowledge on the role of EBV in the pathogenesis of these tumours, and the implications for treatment.

## Introduction

Primary infection with EBV usually occurs via salivary transmission. It is unclear whether the initial infection occurs in epithelial cells or in B cells in mucosal tissues, but it is infection of B cells that enables life-long persistence of the virus as a largely asymptomatic infection [1]. EBV enters resting B cells via the CD21 receptor and MHC-II co-receptor surface molecules [2-4]. In vitro, infection of B cells results in expression of a limited subset of genes which cooperate to induce cell proliferation and transformation into lymphoblastoid cell lines; these genes include six nuclear antigens (EBNA1, EBNA2, EBNA3A, EBNA3B, EBNA3C and EBNA-LP) and three membrane proteins (LMP1, LMP2A and LMP2B) which are expressed together with abundant non-coding RNAs (EBER1 and EBER2) and several micro-RNAs [5]. In vivo, EBV-infected B cells may undergo limited expansion induced by the transformation-associated viral genes, but thereafter the infected B cells revert to a latent state in the circulating memory B cell pool to evade virus-specific immune T cell responses [1,6]. Normal plasmacytoid differentiation of virus-carrying B cells in lymphoid tissues may lead to reactivation of virus into lytic replication [7], which involves expression of around 80 viral genes and the production of new infectious virions [8]. Released virions may in turn infect epithelial cells in the oropharynx [9-12], facilitating further virus production in differentiating epithelium and release into the oropharynx [13] for horizontal transmission to new hosts.

From this understanding of the normal life cycle of EBV, it is possible to envisage how genetic accidents might give rise to EBV-associated malignancies of B cell or epithelial cell origin [1]. What this classical model of the EBV life-cycle does not explain is how EBV-associated diseases of T or NK cells might arise. Indeed, as mature T and NK cells do not express CD21 it is unclear how these cells become infected in the first place. However, EBV-carrying T and NK cells can undoubtedly result in severe clinical syndromes.

## EBV infection of T or NK cells *in vivo*

EBV is not detected in NK or T cells in the blood of healthy carriers, but may be detected at extremely low frequency in tonsillar NK or T cells [14], notably in some patients with infectious mononucleosis (IM), a self-limiting clinical manifestation of primary EBV infection [1]. Infection of NK or T cells is probably an inefficient and rare event, consistent with the lack of CD21 expression on these cells. Nevertheless, a number of EBV-associated NK and T lymphoproliferations have been identified, and are now recognised to comprise a heterogeneous spectrum of diseases, affecting humans through all stages of life and conferring considerable morbidity and mortality. The fundamental unifying feature of such illnesses appears to be the clonal expansion of EBV-infected T or NK cells, although the specific viral and host factors initiating and potentiating the disease processes remain largely unresolved. Furthermore it remains quite unclear why infection of similar or identical cell types is associated with such a diverse spectrum of clinical illnesses, occurring both in previously EBV-naive and ostensibly EBV-immune individuals. The clinical, pathological and biological features of the individual diseases are detailed below.

## Chronic Active EBV

In 1948, Isaacs described a cohort of patients with fatigue, fever, splenomegaly and small volume lymphadenopathy persisting for 3 months to over 4 years after an initial episode of clinically-defined IM [15]. A subsequent study of acute IM patients noted that although most patients had an unremarkable clinical course, a subset of patients experienced protracted symptomatology over periods of 4 to 28 months; these patients tended to have unusually high and persistent titres of antibodies to EBV capsid antigen (VCA) and a delayed antibody responses to early antigen (EA) [16].

There are now numerous reports of patients with clinical syndromes consistent with 'chronic symptomatic EBV infection', although terminological inconsistencies have caused some confusion. The term chronic active Epstein-Barr virus (CAEBV) disease describes patients with a systemic EBV-positive lymphoproliferative disease characterised by fever, lymphadenopathy and splenomegaly developing after primary EBV infection in patients without known immunodeficiency [17]. Suggested diagnostic guidelines required persistence of symptoms for at least 6 months associated with high IgG antibody titres to VCA and EA. Absent or low titres of antibodies to EBV nuclear antigen-1 (EBNA1) are also characteristic of patients with chronic symptoms following proven IM [17,18]. This definition [17] arose from observations on affected children from the Western hemisphere and was proposed to result from progressive EBV infection of B cells, although this was not formally proven.

### Infection of T and NK cells in CAEBV

The first evidence of an association of CAEBV with infection of non-B cells arose from a detailed clinicopathological study of a young child with clinical and serological evidence of CAEBV [19]. EBNA^+ ^cells were detected in blood, bone marrow and lymph node and, unexpectedly, clonal EBV genomes were identified in peripheral blood CD4^+ ^T lymphocytes. There followed a series of reports, predominantly from Japan and East Asia, demonstrating the striking pathological feature of EBV-infected T or NK cells in the blood or tissue of affected patients [20-26]. Importantly, Southern blot analysis of viral terminal repeats consistently demonstrated clonal or oligoclonal EBV genomes [20-25], implicating the virus in the early stages of disease pathogenesis.

The application of quantitative PCR for EBV genome load in peripheral blood and tissue biopsies [27] has provided a more sensitive diagnostic parameter than EBV serology data, which can be normal in a minority of patients with clear clinicopathological evidence of CAEBV [25]. CAEBV patients assessed prior to therapy have viral load values in the order of 10^3^-10^7 ^genomes/10^6 ^PBMC [25,28] and 10^2^-10^6 ^copies per ml of plasma [29-31]. There is evidence to suggest that disease severity correlates with higher viral loads [25].

Interestingly, a very recent study of CAEBV cases in US patients (of predominantly non-Asian descent) found that, in contrast to the East Asian data, B cells were the major target of EBV, with clonality demonstrable in all cases. Cases with T and NK disease did occur, albeit less frequently [32]. Reliable estimates of the incidence of CAEBV cannot be made from existing published data, but the indications are that it is rare in East Asia and even rarer in the West [33].

### Clinical features, prognosis and therapy for CAEBV

A detailed Japanese study of 30 CAEBV cases [25], followed by a nationwide survey that captured data on 82 patients in the period 1990-2001 [28], included elevated EBV DNA load in blood or tissue as a diagnostic criterion alongside the increasingly recognised clinical features, including: fever, hepatitis, lymphadenopathy, hepatosplenomegaly, pancytopenia, uveitis, interstitial pneumonia, hydroa vacciniforme [34], or hypersensitivity to mosquito bites [22]. Interestingly, patients in these analyses could be delineated into two groups according to whether T cells or NK cells were the predominant EBV-harbouring cell, and each respective group appeared to exhibit different clinical features and prognosis. T cell-type infection was characterised by fever and high titres of EBV-specific antibodies, whereas patients with NK cell-type infection exhibited hypersensitivity to mosquito bites and high titres of IgE as distinguishing features. Patients with T cell-type infection appeared to have significantly poorer outcomes [25,28,35]. For the whole study group of 82 CAEBV patients, predominantly children, overall survival was 58% at 10 years [28].

The optimal treatment for CAEBV remains unclear but given the generally poor outcomes following immunoregulatory drugs and antiviral agents, a new therapeutic approach comprising sequential immunomodulation, cytotoxic chemotherapy and allogeneic hematopoietic SCT (allo-HSCT) has been studied [36]. In a small cohort of 18 patients receiving such a protocol, the 3-year overall survival was 95.0%. Importantly, a plateau in the survival curve is evident, suggesting the potential of allo-HSCT to achieve long-term disease-free survival for patients with severe CAEBV.

The recent retrospective US study described similar clinical features, albeit in an older cohort (mean age 19 years) and comparable survival data, with no apparent difference in outcome between those with T or NK disease and B cell CAEBV cases. Allo-HSCT appeared to provide a curative option for some patients, sometimes in the context of refractory disease [32].

## EBV-associated Haemophagocytic Lymphohistiocytosis

**Figure 1:**
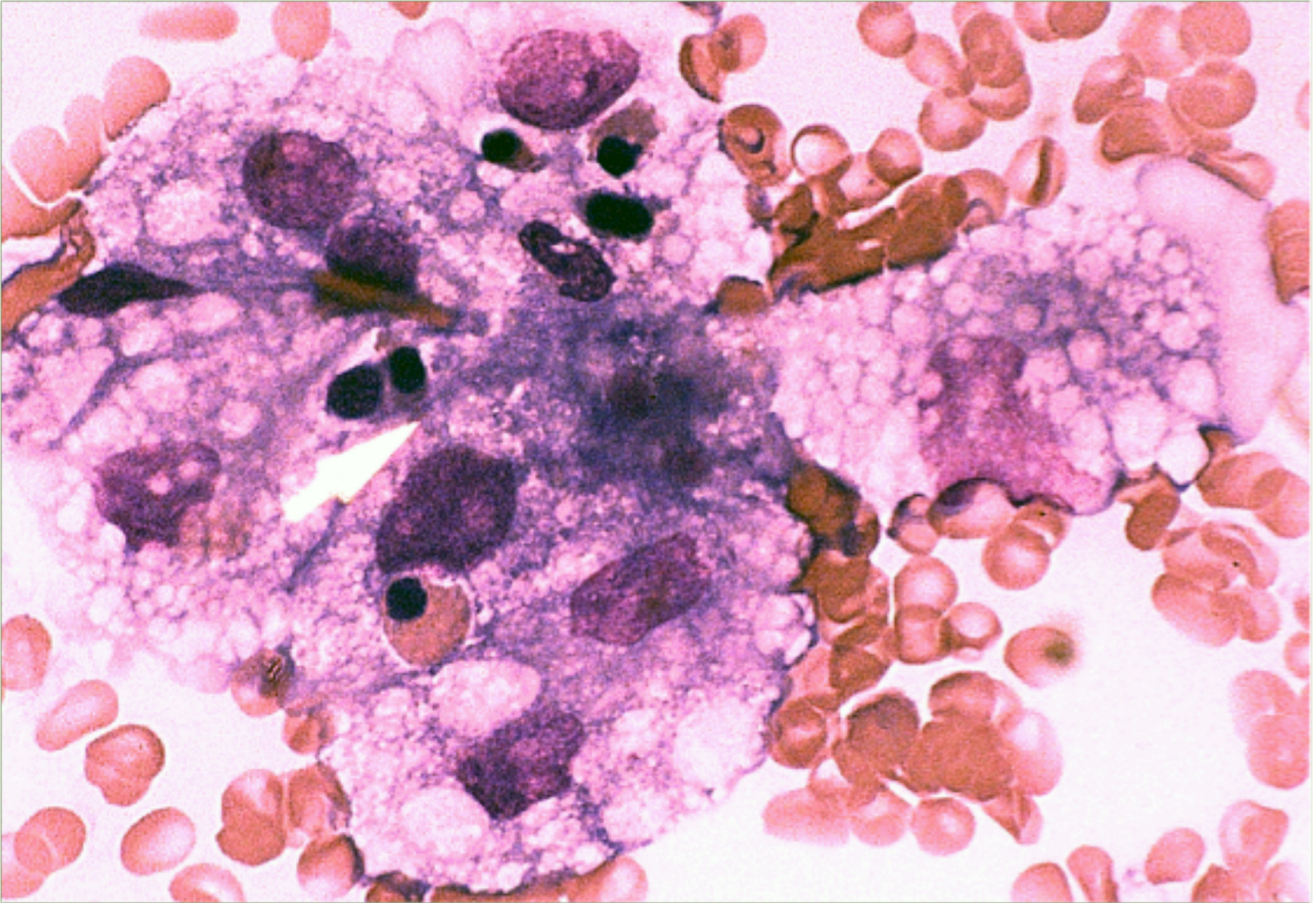
**Haemophagocytosis in the bone marrow**. A photomicrograph of a bone marrow aspirate (original magnification x200) from a UK patient, showing an area of marked macrophage activity and haemophagocytosis. Lipid-laden macrophages are seen to engulf haemopoeitic precursors.

The earliest description of a distinct clinical syndrome associated with histological evidence of erythrophagocytosis (see Figure 1) was reported in 1939 by two Oxford Pathologists [37]. A detailed description of four fatal cases, termed 'histiocytic medullary histiocytosis' was summarised as follows. *"These cases illustrate what we have come to regard as the typical clinical course of the disease: fever, wasting and generalised lymphadenopathy are associated with splenic and hepatic enlargement and in the final stages jaundice, purpura and anaemia with profound leukopenia may occur. Post mortem examination shows a systematised hyperplasia of histiocytes actively engaged in phagocytosis of erythrocytes"*. These prescient observations remain central features of internationally agreed diagnostic criteria of the present-day clinicopathological syndrome now termed Haemophagocytic Lymphohistiocytosis (HLH) [38].

Similar clinicopathological entities have been reported under various terminologies, including familial haemophagocytic reticulosis [39], familial erythrophagocytic lymphohisticytosis [40], histiocytic medullary reticulosis [41] and malignant histiocytosis [42]. These reports included seemingly inherited syndromes and some attributable to, or associated with, malignancy. One Japanese survey estimated that the incidence of HLH was less than 1 case/million population/year [43], although the diagnostic challenges and the nature of this study suggest that this may be an underestimate. The incidence of HLH in the Japan is likely to be higher than that in Western countries.

### EBV as an aetiological agent of a subset of HLH

A possible link with viral infection was highlighted from a study of HLH in a group including patients immunosuppressed following renal transplantation [44]. Evidence of active viral infection, predominantly herpesviruses, was obtained in the majority of cases. This entity was termed virus-associated haemophagocytic syndrome (VAHS). Two cases with high IgM titres to EBV, suggested a role for EBV in disease pathogenesis in some cases of HLH [44]. The apparent association with primary EBV infection prompted a detailed analysis of 52 cases of fatal IM in which, following a initially typical presentation of IM, severe pancytopenia developed together with bone marrow evidence of infiltration by lymphoid cells, cellular necrosis and marked histiocytic haemophagocytosis. The median survival time of these patients was six weeks, in whom an Epstein-Barr virus-associated haemophagocytic syndrome was strongly implicated in the cause of death [45]. It is now clear that EBV is the most frequent cause of acquired HLH in the immunocompetent host [43,46,47].

A major breakthrough implicating EBV as a key aetiological agent in HLH, was the identification of viral genomes within T lymphocytes in tissue biopsies of affected individuals [48-50]. Monoclonality of EBV by Southern blot analyses together with clonal rearrangements of T-cell receptor gene sequences provided evidence of clonal proliferation of a T cell infected with EBV. This was supported by *in situ *hybridisation analyses at the single-cell level, showing EBER-specific signals exclusively within a CD45RO^+ ^TCRβ^+^population; importantly, EBERs were not detectable in B cells or macrophages [51]. Notably, some reports indicated that EBV^+^T cell lymphoma could arise from or co-exist with HLH [48], although clear delineation between the two entities is not always straightforward [52-54].

The majority of published data have confirmed that CD3^+ ^T cells, most often the CD8^+ ^subset, both in tissue biopsies [21,49,51,52,55-57] and circulating lymphocytes [26,58], are the dominant infected population in EBV-HLH. However, unequivocal infection of NK cells has also been observed, and may even be the dominant infected cell type in some patients [59].

### Epidemiology and risk factors for HLH

The majority of cases of EBV-HLH occur in the context of primary infection [45,49,50,60] in children and adolescents [61-63]. Adult cases of HLH are rare and are more frequently attributable to malignancy, particularly lymphoma [43,64]. In common with other entities within the spectrum of EBV^+ ^T and NK lymphoproliferations, the literature is dominated by a majority of EBV-HLH cases reported by study groups in East Asia [62]. However, instances in patients of European [49,58], Middle Eastern [54], North American and Hispanic [52] ethnicities have been described. Notwithstanding the fact that inherited immunodeficiencies [65-69] strongly predispose to HLH, and that the original description of VAHS arose in a largely immunocompromised patient group [44], most patients presenting with EBV-HLH have no clinical history of immunodeficiency.

### Pathophysiology of HLH

An in vivo model of HLH [70] described the emergence of erythrocyte and platelet antibodies at the time of peak viral load. This appearance of antibody-coated erythrocytes anteceded erythrophagocytosis in tissues and heralded the onset of the full clinical syndrome. This phagocytosis was shown to be specifically mediated by Fc-mediated macrophage activation and results in the observed cytopenias.

The clinical manifestations of HLH are, at least in part, a result of a dramatically dysregulated inflammatory response due to release of pro-inflammatory cytokines including IFN-γ, TNF-α, IL-6, IL-10 and M-CSF [71]. These mediators are secreted by activated T-lymphocytes and infiltrating histiocytes, which can instigate tissue necrosis and organ dysfunction. Inflammatory cytokines are also responsible for the haematological and biochemical manifestations such as cytopenias, coagulopathy and elevated triglycerides [65]. Serum Fas ligand (a membrane protein expressed by cytotoxic T and NK cells) has also been noted to be elevated in patients with HLH [72] and may explain features such as liver dysfunction.

Impairment of the cytotoxic function of NK and T cells seems to be a common denominator across both inherited and acquired HLH syndromes [47,73], although the mechanisms leading to cytolytic defects in immunocompetent patients with EBV-HLH are not clear. Elevated levels of cytokines such as IL-12 have been shown to impact on NK function [74]. The apparent geographical disparities in the incidence of EBV-HLH may suggest a hitherto unidentified genetic susceptibility resulting in a dysfunctional immune response to infected cells.

### Prognosis and therapy of EBV-HLH

The aims of therapy are to suppress the augmented inflammatory response with immunosuppressive/immunomodulatory agents, support and restore organ function, and eliminate EBV-harbouring cells with cytotoxic drugs [75-81]. The best clinical evidence has followed from studies based on the international HLH-94 protocol [38,82,83], incorporating etoposide, dexamethasone and Cyclosporin A [82]. In the case of refractory disease [63], or those with familial defects [84], allo-HSCT can result in long-term disease-free survival.

An analysis of 78 children with EBV-HLH treated with an etoposide-based regimen showed 75.6% of patients were alive and well after a median follow-up of 4 years, indicating the effectiveness of similar immuno-chemotherapy protocols as used for familial disease [38,82]. The prognosis and outcome of adult patients with EBV-HLH has been less well studied. The available published data [52,54,64,85] suggest a more adverse prognosis for adults than children with EBV-HLH, although a higher incidence of co-existing lymphoma in adults, and lack of treatment uniformity may confound these data.

## Extra-nodal NK/T-cell lymphoma, nasal type

Extra-nodal NK/T-cell lymphoma (ENKTL) is a relatively recently characterised clinicopathological entity, being formally incorporated into the WHO classification of haematopoietic and lymphoid tumours in 1999 [86]. However, this entity was probably recognised over a century earlier in 1897 by McBride, who described a patient in whom an ulcer developed on the left lateral surface of the nose that within a year, at the time of death, had extended to both cheeks causing extensive tissue damage to the nose and upper lip [87]. A subsequent report in 1921 [88] described two patients with destructive nasal lesions, in whom syphilis was excluded as a diagnosis and no infectious organism could be identified. Further clinical and histological accounts of such a disease accumulated over the following decades [89-95]. Various terminologies for ENKTL have been used, including: lethal midline granuloma, rhinitis gangrenosa progressiva, polymorphic reticulosis, and malignant midline reticulosis.

### Phenotype and genotype of ENKTL

A study by Ishii *et al *was the first to demonstrate that the malignant cells in ENKTL reacted with anti-sera directed to T cell, but not B cell, antigens [96]. A separate analysis found evidence of rearranged T cell receptor (TCR) genes in ENKTL tissue indicative of a clonal T-cell proliferation [97]. The apparent T-cell origin of this lymphoma was corroborated by further pathological studies in East Asia [98,99] and the United States [100]. However, the development of antibodies against the CD56 antigen questioned the T cell phenotype of the malignant cells [101-104]. It is now recognised that the majority of ENKTL tumours are of NK cell origin, with germline T cell receptor gene configurations [102,105-110]. The reactivity of polyclonal anti-CD3 antibodies with the cytoplasmic subunit (ε-chain) of the CD3 molecule in formalin-fixed tissues is the likely explanation for the original phenotypic interpretation [111-114].

The characteristic phenotype of ENKTL is now understood to comprise CD2^+^, CD56^+^, surface CD3^- ^(as demonstrated on fresh/frozen tissue) and cytoplasmic CD3ε^+ ^(as demonstrated on FFPE tissues). The largest clinicopathological study of ENKTL since its incorporation into the WHO classification [86,115], analysed 136 cases of ENKTL and confirmed the expression of CD56 and cytotoxic markers (TIA-1 and granzyme) in a majority of cases, but also identified a minority of tumours (14%) with a CD8^+ ^phenotype. Rearranged T cell receptor genes were found in approximately one-third of 52 cases tested [116].

### Association of ENKTL with EBV

The first compelling evidence implicating EBV in the development of T and NK lymphomas arose from a report describing 3 patients with clinical and serological features suggesting pre-existing CAEBV, who subsequently developed fatal T cell lymphoma containing clonal EBV [117]. Subsequent studies identified EBV genomes and/or EBERs within the tumour cells of both nasal and extra-nasal T and NK lymphomas arising in children and adults [103,107,109,118,119]. The association with EBV was observed to be most robust in extra-nodal lymphomas and those arising in the nasopharynx [120]. The clonal and episomal form of the virus in the tumour cells [121,122], together with the expression of EBV-encoded transcripts and proteins [21,121-124], suggested a causative role for the virus in disease pathogenesis. The association of ENKTL with EBV is invariable, irrespective of geographical origin [116]. Indeed, demonstration of the virus in the malignant cells is virtually a requisite for diagnosis [115].

### Epidemiology and clinical features of ENKTL

ENKTL is an aggressive malignancy with a unique geographical distribution; rare in Western countries and more frequently encountered in East Asia and Central/South America [116,125-131]. Robust data are lacking for the incidence of ENKTL as defined by WHO diagnostic criteria, and this is currently being addressed by the ongoing International T cell project directed by Massimo Federico, Modena, Italy (ClinicalTrials.gov Identifier: NCT00705809). Nevertheless, large epidemiological studies of consecutive NHL cases in China [127] and Korea [126] have shown that whilst mature T and NK neoplasms (of all subtypes) comprise approximately 30% NHL, ENKTL accounts for approximately 4-6% of incident NHL cases. By contrast, the best estimate of incidence in Europe and the United States is that ENKTL represents 4% of all NK and T cell lymphoma subtypes [116], which equates to approximately 0.5% of all NHLs [132]. From these data it can be estimated that the incidence of ENKTL may be less than 0.5 cases/million population/year in Western countries and in the region of 2-4 cases/million population/year in some Eastern regions.

Patients with ENKTL are usually immunocompetent. The median age of presentation is 45-50 years with a male: female ratio of 2-3:1 [116,126,133-135]. However, it should be emphasised that demographic data on this disease (relating to incidence, age/sex distribution and patient ethnicity) is not well characterised outside East Asia.

ENKTL commonly affects the upper aero-digestive tract (characteristically the nasal cavity), though extra-nasal disease (e.g. skin, gastrointestinal tract, testis) can account for one quarter of cases [116]. Primary lymph node involvement is rare. Bone marrow involvement at diagnosis, as determined by conventional immunohistochemistry, occurs in a minority (6-14%) of cases [116,136] although this may be an underestimate [137]. Clinical presentation is typically referable to local symptoms from a nasal mass, including obstructive symptoms and bleeding. Hoarseness of voice, dysphagia, proptosis, ophthalmoplegia and dysphonia can also occur according to the extent of local tumour invasion [138]. Poorer prognosis is conferred by local invasiveness, elevated serum lactate dehydrogenase, advanced stage disease and the presence of B symptoms [116,136].

### Therapy for ENKTL

ENKTL is clinically aggressive, displaying inherent resistance to anthracycline-based chemotherapy regimens such as CHOP [139], adopted empirically from B cell lymphoma studies. The outcome of extra-nasal and advanced stage disease is extremely poor [116,136,138]. However, the tumours are usually sensitive to radiation therapy which, when given at relatively high doses [140], is the mainstay of front-line therapy for localised disease [141]. However, in spite of high rates of initial response following involved-field radiotherapy, up to 50% of those with localised disease will experience relapse, usually within a year of completing first-line therapy [138,141-143]. Recently published data from early phase clinical trials examining concurrent chemo-radiotherapy in localised ENKTL [144,145], and chemotherapy combined with L-asparaginase for relapsed/refractory ENKTL are encouraging [146-149], but remain to be tested in randomised phase III clinical studies. Nevertheless, despite signs of therapeutic progress, an extremely poor outcome is anticipated for the majority of patients with this disease. Data from the international T cell project study [116], showed a median overall survival of 7.8 months for patients with ENKTL, representing the poorest survival of all T cell lymphoma subtypes examined [150].

Attempts to improve outcome in ENKTL have included studies of high-dose chemotherapy (HDT) with autologous stem cell transplantation (ASCT), primarily undertaken in East Asia. The majority of published data are based on retrospective analyses of relatively small cohorts [151-153]. A pooled analysis of 47 patients from 3 studies suggested a survival advantage for those undergoing ASCT, although the survival benefit appeared small and larger collaborative studies are necessary to demonstrate unequivocal benefit for this approach.

The notion of harnessing a graft-versus-lymphoma effect against malignancies inherently resistant to conventional therapies is attractive and clearly has a role for some patients with more common subtypes of T cell lymphomas [154,155]. The invariable presence of EBV in the tumour cells of ENKTL, expressing the viral antigens EBNA1, LMP1 and LMP2, provides additional allo-reactive T cell targets. Moreover, initial *in vivo *studies have adoptively transferred autologous, *ex-vivo-*stimulated, LMP2-specific cytotoxic T lymphocytes to patients with ENKTL with encouraging results [156,157].

The role of allogeneic HSCT for patients with ENKTL remains unclear. Data from two small series [153,158] (comprising six and twenty-two patients) suggest that a proportion of patients with relapsed and refractory disease can achieve long-term disease-free survival, presumably mediated through a graft-versus lymphoma effect.

## Aggressive NK leukaemia

The first distinct report of an aggressive NK cell leukaemia (ANKL) in an adult described in a 71 year old white man from the United States [159], although the majority of subsequent reports from East Asia have occurred in younger individuals [160]. A cell line established from the first case retained morphological, immunologic, and functional characteristics of NK cells [159]. Further reports of clinically aggressive leukaemias displaying neither a B nor T cell phenotype [102,159,161,162] suggested that such malignancies can arise from non-T cell large granular lymphocytes, or NK cells. The WHO subsequently recognised this aggressive leukaemia as a distinct clinicopathological entity and ANKL was separately incorporated into the lymphoid tumour classification [86].

### Association of EBV with ANKL

In the initial reports of ANKL, studies for EBV were not always performed, although it is now recognised that > 90% of ANKL cases harbour clonal, episomal EBV [23,163,164].

### Clinical features, prognosis and therapy for ANKL

ANKL is extremely rare, with approximately 100 published cases worldwide [165]. The disease typically affects young to middle age patients (mean age approximately 40 years), with a slight male predominance. Patients with ANKL are almost always systemically unwell at presentation, usually with a high fever and constitutional symptoms such as sweats and weight loss. A leukaemic picture is invariably found, associated with prominent thrombocytopenia and variable degrees of anaemia and neutropenia [165]. Cytogenetic abnormalities are seen in at least two-thirds of cases and are often complex [160,165].

Most cases of ANKL pursue an inexorable clinical course, typically displaying resistance to cytotoxic therapies. Complications such as coagulopathy, haemophagocytic syndrome and multi-organ failure are not uncommon. In spite of treatment with intensive chemotherapy, mortality from ANKL is virtually inevitable with a median survival of less than 2 months [160,163,166-168]. Even for the minority of patients who experience an initial remission following an anthracycline-based regimen, relapse occurs invariably and attempts to improve outcome by the use of allogeneic bone marrow transplantation [169] have not proved widely successful [168].

### Differences between ANKL and ENKTL

ANKL shares many features with extra-nodal NK/T lymphoma, including: cytological features, an almost identical immunophenotype [108] (CD2^+^, CD3ε^+ ^and CD56^+^) although CD16 is thought to be more frequently expressed on ANKL than ENKTL [168,170], and a lack TCR gene rearrangements [108,165]. As with ENKTL, ANKL is also seen with increased incidence in East Asia. In the vast majority of cases, distinct clinical features allow these diseases to be clearly delineated, although some patients with ENKTL may progress to an aggressive, systemic disease akin to ANKL [171,172]. However, comparative genomic array studies support the notion that ANKL and ENKTL are distinct entities [173].

## EBV gene expression in T and NK lymphoproliferations

**Figure 2:**
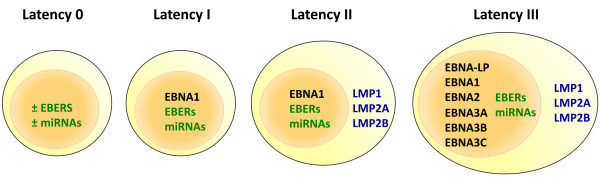
**Patterns of latent viral gene expression in EBV-associated tumours**. Schematic illustrating major patterns of EBV gene expression observed in different virus:host interactions; non-coding RNAs and micro-RNAs are indicated in green type, nuclear proteins in black type, and membrane proteins in blue type. Latency 0, sometimes referred to as '*in vivo *latency, is the type of latency observed in non-dividing circulating memory B cells of healthy carriers; it is possible that the majority of these cells express no viral genes at all, but that a minority may express non-coding RNAs. Latency I was originally identified in Burkitt' lymphoma, Latency II in nasopharyngeal carcinoma and Hodgkin's lymphoma, and Latency III in post-transplant lymphoproliferative disease.

A key element to understanding the possible role of EBV in associated disease is knowledge of the pattern of viral gene expression. As mentioned earlier, EBV is a potent transforming agent for primary B cells in vitro, where the establishment of lymphoblastoid cell lines requires the cooperative functions of several so-called 'latent genes' [1]. The pattern of viral gene expression in LCLs is commonly referred to as 'Latency III' [5,174]. In vivo, Latency III may be observed in some EBV infected tonsillar B cells in acute IM patients [14,175] or in immunoblastic B lymphomas in iatrogenically immunosuppressed patients [176,177]. However, EBV-associated malignancies are usually associated with more restricted patterns of viral gene expression (Figure 2), because other cellular genetic changes negate the requirement for full Latency III expression and because expression of viral antigens for initiating or maintaining cellular transformation has to be balanced against the cost of antigen exposure to immune-surveillance mechanisms.

The first EBV malignancy shown to express a more restricted pattern of latent viral gene expression was Burkitt's lymphoma (BL), where the only viral protein to be expressed is EBNA1 [178,179] which is essential for the maintenance of the viral episome in dividing cells [180-182]. These tumours also express the non-coding EBERs [178] and several microRNAs derived from the BART transcripts [183]. This pattern of gene expression is commonly referred to as Latency I (Figure 2). Such a restricted pattern of transformation-associated latent genes is possible in BL as these tumours invariably carry chromosome translocations resulting in deregulated expression of the *c-myc *oncogene [1,184]. It has been proposed that EBNA1, EBERs and BARTs might cooperate with *c-myc *driven proliferation in maintaining the malignant phenotype by contributing anti-apoptotic and immune-modulating functions [184].

A third major type of latency in EBV-associated malignancies is Latency-II, in which LMP1, LMP2A and LMP2B proteins are expressed in addition to the Latency I genes (Figure 2). These membrane proteins are important modulators of cell signalling, conferring strong protection against apoptotic signals [185,186] and blocking terminal differentiation of infected cells [187-190]. Prototypic examples of Latency II tumours are nasopharyngeal carcinoma and Hodgkin's lymphoma [1].

Reverse Transcriptase Polymerase Chain Reaction (RT-PCR) assays for EBV latent transcripts can be very sensitive and, by taking advantage of the fact that all latent protein mRNA are products of spliced primary RNA transcripts [8], can be made highly specific and eliminate the possibility of contamination of the assay with viral DNA. In contrast to immunohistochemistry and in situ hybridisation techniques, RT-PCR assays only provide information at the population level within a sample and heterogeneity within a tumour may therefore be missed. However, as EBNA1 is produced from different promoters with different splice products in Latency I or Latency II (Qp promoter) and Latency III (Cp or Wp promoters), and expression of LMP1/2 can distinguish between Latency II and Latency I, RT-PCR can be a simple and sensitive method for distinguishing the major forms of latency [5]. A caveat, however, is that RT-PCR detects expression in the total population which may contain heterogenous patterns of latency at the single cell level.

It is important to recognise that the Latency I, II, III nomenclature represents just three common snapshots of gene expression over a spectrum ranging from Latency 0 (no EBV antigen expression, as observed in circulating memory B cells in healthy infected individuals), to the Latency III observed in EBV-transformed lymphoblastoid B cell lines. Some tumours may not fall neatly into one of these patterns of latency; for example, LMP1 is often poorly expressed or undetectable in nasopharyngeal carcinomas which otherwise display a Latency II phenotype. Furthermore, immunohistochemical analysis may indicate heterogeneity of expression within a single biopsy, but the pattern of latency is often misleadingly described according the sum total of viral gene products detected.

Against this background, what is known about the pattern of viral gene expression in EBV-associated T and NK cell diseases?

### EBV gene expression in CAEBV

Analyses of EBV-encoded proteins in cell lines established from patients with CAEBV [191,192], suggest a Latency II pattern of viral gene expression. Studies examining viral gene expression in *ex-vivo *lymphocytes from CAEBV patients have been scarce. Iwata *et al *[193] recently described a pattern of EBV latent antigen expression including Qp-initiated EBNA1, LMP1, and LMP2; EBNA2 and lytic transcripts were absent. This pattern is indicative of Latency II. Although this study used RNA extracted from total PBMC, the virus in each case was confirmed to be predominantly within the T or NK population. These results were consistent with previous, non-quantitative PCR studies on CAEBV PBMC *ex-vivo *[35,194].

### EBV gene expression in HLH

The pattern of EBV gene expression in EBV-HLH remains unclear. EBERs are frequently expressed [51]. However, whether EBV-HLH lymphoproliferations display a Latency II pattern of viral gene expression, in keeping with the related T or NK disease CAEBV [195] has not been adequately studied. One limited study analysed mRNA from splenic and peripheral blood mononuclear cells in 3 patients with EBV-HLH and found expression of EBERs, Wp/Cp- and Qp-initiated EBNA1, together with EBNA2, LMP1 and LMP2A transcripts [196]. This suggests alternate promoter usage in different cell populations, most likely with a Latency III expression in B cells, which limits interpretation of the data. In another study [59] EBER transcripts were detected in the absence of protein-coding transcripts, which is at odds with the requirement for EBNA1 expression for maintenance of the viral genome in dividing cells. Further studies, preferably including analysis of expression at the single cell level, are required to ascertain the pattern of viral gene expression typically exhibited in EBV-HLH.

### EBV gene expression in ENKTL

Initial analyses of six cases of ENKTL, confirming the presence of the virus within the malignant cells [121], also found numerous LMP1 positive cells by immunohistochemical staining in four of six cases. A more comprehensive follow-up study analysed 23 cases of ENKTL [123]. Immunostaining for LMP1 revealed heterogeneous membrane positivity in a sub-population of EBER^+ ^cells in 15 of 23 cases. Of note, both cases of extra-nasal tumours analysed by Chiang *et al *[123] were LMP1 negative and, importantly, none of the 23 cases expressed EBNA-2 or BZLF1 protein, which is consistent with Latency II or Latency I and no activation of lytic cycle. Further characterisation of RNA transcripts by non-quantitative end-point RT-PCR revealed expression of BARTs (precursor transcripts for BART miRNAs) in the majority of cases, whilst EBNA1 transcripts were detected in 15/23 cases and confirmed to be Qp-initiated. LMP1 transcripts were readily detected in all cases, although LMP2A and LMP2B mRNAs were absent or low in the majority of tumours.

**Figure 3:**
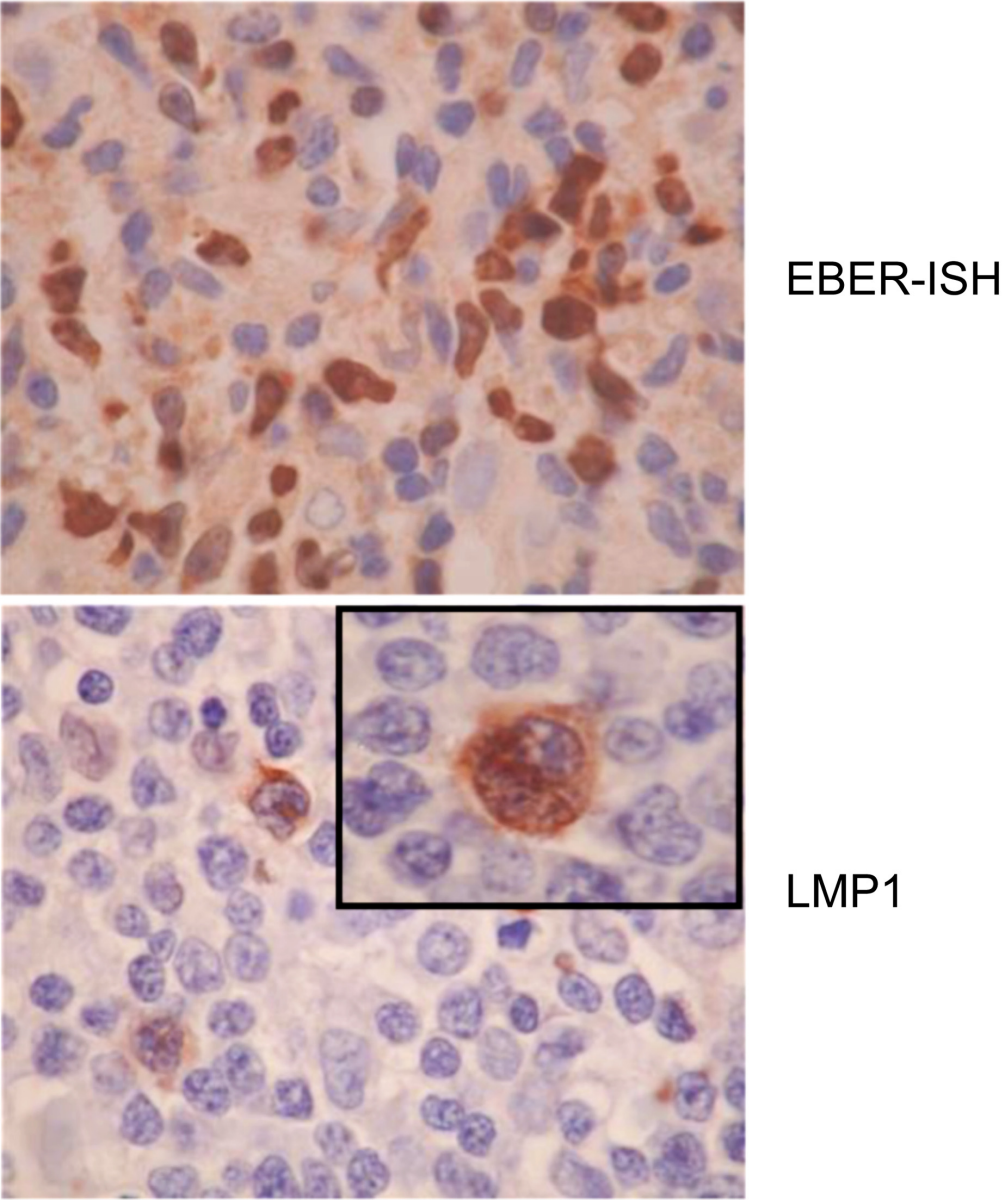
**Heterogeneous LMP1 expression within an ENKTL tumour**. Upper photomicrograph: EBER in-situ hybridisation on a 4 μm formalin-fixed, paraffin-embedded ENKTL tissue section (optical magnification x200). Courtesy of Dr Simon O'Connor, Department of Histopathology, Nottingham University Hospitals. Lower photomicrograph: Immunohistochemical stain of a 4 μm formalin-fixed, paraffin-embedded ENKTL tissue section using CS1-4 (anti-LMP1) antibodies. The image was captured with a Nikon CoolpixE995 digital camera, via a Nikon Eclipse E400 microscope (optical magnification, ×400).

These initial data have been broadly supported by subsequent studies [21,122-124,197-200] suggesting that ENKTL typically express a Latency II pattern of gene expression, although both inter- and intra-tumour heterogeneity exists. In particular, LMP1 expression is variable and heterogeneous at the single cell level in ENKTL [123,200] (Figure 3), whereas LMP2A and LMP2B mRNA levels appear low or absent in analysed cases [123,197,199]. Recently, we demonstrated that ENKTL express a hitherto unrecognised LMP2 transcript initiated from within the terminal repeats of the EBV genome [191] that is predicted to encode a protein identical to that from LMP2B transcripts. As the LMP2B protein contains most of the immune T cell epitope sequences identified to date for LMP2A/B, the LMP2 product identified in ENKTL represents a viable target for adoptive T cell immunotherapy. Although the viral proteins expressed in ENKTL are not normally immunodominant [1], ongoing work to amplify LMP1-and LMP2-specific responses for adoptive transfer have so far produced encouraging results for the treatment of ENKTL [156,201].

### EBV gene expression in ANKL

Due to the rarity of this disease and the often rapidly fatal course, analyses of EBV gene expression in ANKL have been scarce. However, one study from Shanghai found that nine consecutive cases of EBER^+ ^ANKL were LMP1 negative by immunostaining [165].

## Unanswered questions in EBV-associated NK and T lymphoproliferations

A fundamental unanswered question remains: how does EBV infect NK or T cells? Although experimental infection of primary NK cells and NK cell lines has been reported to be an efficient process [202] these results have not been replicated in other laboratories. The rarity of observed NK and T cell infections in vivo, would favour the interpretation that infection of these cells is a rare event, but with potentially catastrophic clinical consequences. Whilst the mechanisms of infection *in vivo *have yet to be elucidated, two attractive possibilities remain to be excluded. The first is via immunological synapses, whereby conjugates between effector T cells or NK cells and virus-harbouring target cells might in rare circumstances facilitate transfer of virus from the target cell to the immune effector cell, akin to that demonstrated for HIV [203,204]. The second mechanism might involve infection of immature precursor cells. It has previously been reported that immature thymocytes transiently express CD21 and can be infected with EBV and whilst the pattern of viral gene expression was not established, EBV was found to synergise with IL-2 to induce proliferation of these cells [205]. Furthermore, as CD34^+^, CD38^- ^stem cells can be differentiated *in vitro *to transiently express CD21 prior to commitment to T or NK cell lineage (CSL, unpublished observation), this raises the intriguing possibility that infection of a precursor cell has the potential to give rise to either or both NK cells and T cells carrying the same monoclonal EBV episomes. This latter point is relevant to cases of CAEBV and HLH where both cell types may carry EBV in the same patient. Indeed, one recent study on childhood T/NK lymphoproliferations indicated that in some cases the same monoclonal EBV might be present in different cell populations, although no evidence of infection of CD34^+ ^stem cells was found [206].

A second question relates to the heterogeneity of EBV gene expression both between patients and within the same lesion, as is seen with LMP1 expression in ENKTL. Heterogeneity within a lesion may represent two different phenotypes of the same parental tumour. Alternatively, it may reflect a dynamic process akin to that reported in EBV-transformed lymphoblastoid B cell lines, where LMP1 levels vary among individual cells such that the difference between the highest and lowest expressing cells may be as great as 100-1000 fold at any given time, but within hours the minimally expressing cells revert to higher levels of LMP1 [207,208]. Studies on EBV^+ ^T and NK tumour cell lines suggest that LMP1 expression in individual cells within a lesion may be substantially affected by local concentrations of cytokines and by interactions with other cell types [209,210]. Heterogeneity between tumours may also be important; the lack of detectable LMP1 in two extranasal ENKTL from one study [123], raises the possibility that additional cellular genetic aberrations may be driving a more malignant tumour phenotype that no longer requires expression of the LMP1 oncogene. In this context it is notable that ANKLs appear not to express LMP1 [165].

Finally, the recent demonstration that ENKTLs express a novel LMP2 transcript, putatively encoding an immuno sub-dominant LMP2B protein, has implications for both the pathogenesis and therapy of these tumours [191]. These tumours represent the first example of EBV-infected cells naturally expressing LMP2B in the absence of LMP2A. This is notable since the N-terminus of LMP2A, which is lacking in LMP2B, is responsible for its major signalling functions. LMP2B acts as a dominant-negative modulator of LMP2A function [211,212]. This dominant-negative property of LMP2B has hitherto been regarded as its major function. However, its expression in the absence of LMP2A in ENKTL tumours highlights the potential of LMP2B to function independently [212,213]. As LMP2B is likely to contribute to the initiation or potentiation of EBV-associated NK and T cell diseases, characterisation of the independent functions of LMP2B may identify novel targets for therapy.

## Conclusions

The rarity of EBV-associated NK and T cell malignancies, and the consequent difficulty in obtaining patients and tissues for study, has meant that these diseases have been less well-studied compared to their B and epithelial cell counterparts. This is unfortunate since they are clinically challenging and the prognosis for many patients is dismal. The aim of this review was to draw attention to the current state of knowledge of the clinical and virological features of these diseases, and to highlight some unanswered questions regarding the role of EBV in the disease pathogenesis. Deciphering the precise contribution of EBV to these rare T and NK lymphoproliferations will require collaborative, translational efforts to study sufficient numbers of patients and ultimately achieve meaningful therapeutic progress for patients.

## List of Abbreviations

ANKL: Aggressive NK cell leukaemia; CAEBV: Chronic active EBV infection; EA: Early Antigen of EBV; EBER: EBV-encoded RNA; EBNA: EBV-encoded nuclear antigen; EBV: Epstein-Barr virus; ENKTL: Extra-nodal NK-/T-cell lymphoma FFPE: Formalin-fixed paraffin-embeded; HIV: Human immunodeficiency virus; HLH: Haemophagocytic lymphohistiocytosis; HSCT: Haematotopoietic stem cell transplantation; IM: infectious mononucleosis; LMP: EBV-encoded latent membrane protein; NHL: Non-Hodgkin lymphoma; RT-PCR: Reverse transcriptase, Polymerase chain reaction; TCR: T cell receptor; VCA: Viral capsid antigen of EBV; VAHS: virus-associated haemophagocytic syndrome.

## Competing interests

The authors and funding bodies have no competing interests.

## Authors' contributions

CPF, MR and CSL conceived and wrote the manuscript. All authors read and approved the final manuscript.
